# Mitochondrial and chloroplast genome sequences of *Ulva ohnoi*, a green-tide-forming macroalga in the Southern coastal regions of Japan

**DOI:** 10.1080/23802359.2018.1483778

**Published:** 2018-07-10

**Authors:** Shigekatsu Suzuki, Haruyo Yamaguchi, Masanori Hiraoka, Masanobu Kawachi

**Affiliations:** a Center for Environmental Biology and Ecosystem Studies, National Institute for Environmental Studies, Ibaraki, Japan;; b Usa Marine Biological Institute, Kochi University, Kochi, Japan

**Keywords:** *Ulva ohnoi*, green tide, phylogeny, authentic strain, organellar genome

## Abstract

*Ulva* is a green macroalga often causing a macroalgal bloom, ‘green tide’. *Ulva ohnoi* is a major species composing the green tide of the southern coastal regions of Japan. Here, we sequenced the complete mitochondrial and chloroplast genomes of the authentic strain of *U. ohnoi*. The mitochondrial and chloroplast genomes were of 65,326 bp and 103,313 bp, respectively, and the gene content was highly conserved in the *Ulva* species. The phylogenetic analyses using mitochondrial or chloroplast proteins represented the same topology with high supporting values. These results show that mitochondrial and chloroplast genomes can be used as reliable phylogenetic markers.

*Ulva* is a green macroalga in Ulvophyceae (Verbruggen et al. 2009). Species of *Ulva* are important for some applications, such as food stuffs (Hiraoka and Oka 2008; Holdt and Kraan 2011) and resource of biofuels (Bikker et al. 2016). In ecological aspects, some free-floating species of *Ulva* cause large-scale macroalgal bloom called ‘green tide’ (Fletcher 1996). Particularly, *Ulva ohnoi* is originally the major species composing the green tide in the southern Japanese coasts, such as Tosa bay and Hakata bay (Ohno 1988; Hiraoka, Ohno et al. 2004; Hiraoka, Shimada et al. 2004). In the past two decades, *U. ohnoi* bloom has been observed in Tokyo bay, which may have been introduced from tropical and subtropical waters (Yabe et al. 2009). Because it is difficult to distinguish *Ulva* species by morphological characters (Malta et al. 1999), molecular phylogenetic analyses were used to identify them; however, the complete organellar genomes of authentic strains of *Ulva* have not been reported.

Here, we sequenced the mitochondrial and chloroplast genomes of *U. ohnoi* KU-3321, which is an authentic strain of *U. ohnoi* established through parthenogenesis from subculture of gametes produced by the holotype specimen (strain KA43 in Hiraoka, Ohno et al. 2004). The strain was deposited in the Kobe University Macro-Algal Culture Collection (http://ku-macc.nbrp.jp/locale/change?lang=en). DNA was extracted from 2-week-old thalli. The paired-end libraries were sequenced on the MiSeq sequencing system (Illumina) with the MiSeq Reagent Kit v2 500 cycles. We acquired 9,172,559 read pairs, which were assembled using metaSPAdes v3.11.1 (Nurk et al. 2017). The 65,326-bp and 103,313-bp circular-mapping mitochondrial (AP018695) and chloroplast genomes (AP018696) were obtained, annotated by GeSeq (Tillich et al. 2017), and then manually curated. GC% of the mitochondrial and chloroplast genomes were 34.1% and 25.4%, respectively. The mitochondrial genome possessed 29 conserved protein-coding genes, 7 intronic ORFs, 2 hypothetical proteins, 2 rRNAs and 31 tRNAs. All the protein-coding genes were conserved among *Ulva* sp. UNA00071828 (Melton et al. 2015), *U. pertusa* (Liu et al. 2017), *U. fasciata* (Melton & Lopez-Bautista 2016), *U. prolifera* (Liu and Jun Pang 2015), *U. linza* (Zhou et al. 2016) and *U. flexuosa* (Cai, Wang, Jiang et al. 2017). *U. ohnoi* possessed *trnS* (GCA), but the gene was lacking in the other *Ulva* species. *U. ohnoi* possessed 8 introns; 1 intron each in *atp1*, *cox2*, *nad3*, *rrs*, and *rrl*, and 3 introns in *cox1*. The chloroplast genome possessed 71 conserved protein-coding genes, 7 intronic ORFs, 3 rRNAs and 26 tRNAs. All the genes except intronic ORFs were completely conserved among *Ulva* sp. UNA00071828 (Melton et al. 2015), *U. fasciata* (Melton and Lopez-Bautista 2017), *U. prolifera* (KX342867), *U. linza* (Wang et al. 2017) and *U. flexuosa* (Cai, Wang, Zhou et al. 2017).

Phylogenetic analyses using 29 mitochondrial or 71 chloroplast proteins were performed (Figure 1). Both trees represented the same topology with relatively high bootstrap support, and *U. ohnoi* was the sister of *U. fasciata* as the previous report (Hiraoka, Shimada et al. 2004). These results show that the mitochondrial and chloroplast genomes can be used as reliable phylogenetic markers.

**Figure 1.**
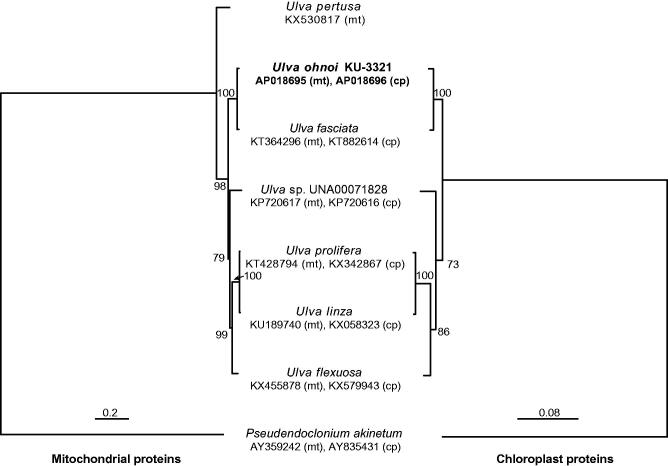
Phylogenetic tree using mitochondrial (left) or chloroplast proteins (right). For mitochondrial tree (left), 8092 amino acids of 29 proteins were used. For chloroplast tree (right), 20,421 amino acids of 71 proteins were used. The amino acids were aligned using Mafft v7.164 (Katoh and Toh 2008), and highly divergent regions were manually trimmed using Mega 7 (Kumar et al. 2016). ML trees were inferred, and non-parametric bootstrapping analyses were performed 200 times using IQ-TREE 1.5.5 (Nguyen et al. 2015). The chloroplast genome of *Ulva pertusa* has been unavailable.
